# Experiences of Parental Suicide-Bereavement: A Longitudinal Qualitative Analysis over Two Years

**DOI:** 10.3390/ijerph18020564

**Published:** 2021-01-11

**Authors:** Lorenza Entilli, Victoria Ross, Diego De Leo, Sabrina Cipolletta, Kairi Kõlves

**Affiliations:** 1 Australian Institute for Suicide Research and Prevention, WHO Collaborating Centre for Research and Training in Suicide Prevention, School of Applied Psychology, Griffith University, Brisbane 4000, Australia; lorenza.entilli@phd.unipd.it (L.E.); victoria.ross@griffith.edu.au (V.R.); d.deleo@griffith.edu.au (D.D.L.); 2 Department of General Psychology, University of Padova, 35100 Padova, Italy; sabrina.cipolletta@unipd.it

**Keywords:** suicide, suicide bereavement, child death, parents, longitudinal qualitative research

## Abstract

Limited research exists on the experiences of parents bereaved by suicide. Our earlier qualitative analysis examined the experiences of parents’ suicide bereavement at 6 and 12 months after their loss. The current study aimed to extend the analysis over 24 months, outlining the key themes of parents’ suicide bereavement experience. In the frames of a longitudinal study of suicide bereavement in Queensland, Australia, parents were interviewed at 6, 12, and 24 months after their loss. Thematic analysis was used to further explore new themes and three key themes identified in earlier analyses: searching for answers and sense-making, coping strategies and support, and finding meaning and purpose. Results at 24 months revealed a clearer differentiation between strategies adopted by mothers and fathers. Anger and blame had changed towards feelings of depression. A polarization was observed between parents still oscillating in brooding rumination and those who have shifted towards sense-making. The former more frequently reported depression symptoms, and the latter reported a more positive attitude towards life and acceptance of their loss. Consistent with the dual-process model, parents managed to reach acceptance after oscillating between sense-making and meaning making. Findings provide insights how suicide loss affects parents, with implications for postvention.

## 1. Introduction

Approximately 800,000 people die by suicide every year [1]. The impact of suicide is far-reaching, with recent estimates indicating that 135 people may be directly exposed to each suicide death [2]. Suicide bereavement can have long lasting impacts, including a higher risk of both mental and physical health problems [3]. Moreover, losing a child to suicide has an extensive impact on parents, who in addition to usual grief reactions are more likely to experience feelings of guilt, responsibility, and stigmatization [4,5].

The literature shows that parents surviving the suicide of a child are among the most heavily impacted groups [6]. It has been noted that parents face a “wall of silence” that hinders the possibility of a dialogue with care professionals, relatives, and the extended social network, all potential resources for coping with traumatic loss [7]. In fact, being able to find an answer to the question of “why” is important for bereaved individuals to reach acceptance [8]. The dual process model of coping with bereavement by Stroebe and Schut [9] suggest that the bereaved often oscillate between sense-making and meaning making before arriving to acceptance, the former referring to the realization and acceptance that the suicide has occurred, the latter to referring to finding a positive response to this event that may lead to post-traumatic growth [10]. Nevertheless, there is a lack of longitudinal studies analyzing individual-level changes in grief reactions after suicide [3,11]. Longitudinal qualitative studies focus on individual narratives and trajectories and can capture critical moments and processes involved in change [12]. Therefore, this approach can be very useful in understanding how parents adjust differently to their loss over an extended period. So far, only a few longitudinal qualitative studies have been conducted on bereaved parents of children who died by cancer [13] or infants who died due to a complex chronic condition [14].

To address this gap in the literature, Kolves and colleagues [11,15] conducted a longitudinal study of bereaved relatives in Australia over two years. The research included a qualitative analysis of the experiences of parents bereaved by suicide at 6 months and 12 months after their loss [16]. The present study aims to extend the longitudinal qualitative study to include a follow-up analysis of the next wave of data—parents’ experiences at 24 months—taking into consideration the overall 2-year window and the 3 separate time observations of parents at 6, 12, and 24 months after the suicide of their child.

## 2. Materials and Methods

This analysis is part of a wider longitudinal study of sudden death bereavement using qualitative and quantitative methods over two years in Queensland, Australia [11,15,16]. In the current paper, only the qualitative results will be analyzed.

People bereaved by suicide were identified through the Queensland Suicide Register, a suicide mortality database. Relatives who had previously provided their consent to be contacted for research purposes were invited to participate in the study approximately five months after their loss. An invitation and information letter were sent via post, and a clinical interviewer followed up two weeks later. If relatives agreed to participate at the time of phone contact, a time and place for the interview was arranged. The study included further follow-up interviews at approximately 12 and 24 months after the loss. For these interviews, participants were sent further invitations at 11 months and 23 months. Interviews in the frames of the pilot study were conducted in 2012, and in the frames of the large-scale study in 2014–2017 [11].

Semi-structured interviews were conducted either by telephone or face-to-face by trained female clinical interviewers with postgraduate health qualifications (psychology, social work, or nursing). Due to length of the study, the interviewers were not necessarily the same for each wave. Nevertheless, interviewers were skilled and experienced in working with the bereaved, proficient in recognizing participants’ needs, and able to provide assistance should a participant become distressed. A short introduction and debriefing were provided to participants at both the commencement and completion of the interview, allowing participants’ concerns, queries, or feelings to be addressed sensitively. The six-month interview incorporated a qualitative component containing open-ended questions about the events leading to the death and relatives’ feelings and experiences since that time; a quantitative component, which included questions about socio-demographic background, medical and psychiatric history (including suicidal behavior) of the deceased and bereaved; and post-event experiences measured with different validated scales [15,16]. The follow-up interviews focused on questions about life events and changes since the previous interview. Initial interviews lasted approximately two and a half hours, and follow-up interviews, two hours. All interviews were audiotaped with the participant’s consent and transcribed verbatim. The Griffith University’s Human Research Ethics Committee (CSR/04/11/HREC) approved all procedures.

Purposive sampling was utilized to select participants from the large-scale study, which included 49 mothers and 24 fathers [16]. Factors considered in sampling included representation of both mothers and fathers, gender of the child, age of parent and child and help seeking. Cases were included until data saturation was reached as for our earlier analysis [16]. The sample of our earlier analyses included seven mothers (Mage = 60.1 years, range: 50–78) and seven fathers (Mage = 59.9 years, range: 50–68). Ten parents were bereaved of their sons and four were bereaved of daughters (Mage = 29.3 years; range: 15–51). One parent of each deceased child was included in the sample (i.e., there were no couples). All of them participated at both 6- and 12-months; however, since then, three participants dropped out, and they were not contactable for the 24-month interview. These were two mothers and one father; one lost a daughter and two a son. Despite the dropouts, the sample was deemed to be still representing mothers and fathers, gender of the child, age of parent and child. More specifically, the parents for the 24-month analysis included five mothers (Mage = 63.2 years, range: 50–78) and six fathers (Mage = 62.3 years, range: 56–68). Eight parents were bereaved of their sons and three of their daughters (Mage = 30.9 years; range: 15–51).

A deductive approach was applied to thematically analyze data obtained at 24 months within the three existing themes identified from the analysis from 6 and 12 months: searching for answers and sense making; coping strategies and support; and finding meaning and purpose [16]. A comparison was conducted between these themes in order to capture thematic changes and group patterns over time. The five-step process [17] for conducting thematic analysis was followed utilizing ATLAS.T8 (Windows). Initial coding was carried out by LE (psychologist); and successive steps into a deeper analysis and grouping were continuously discussed with KK and VR (respectively, a sociologist and a psychologist, both authors of the previous study and whom conducted the earlier analysis). The previous coding experience allowed a meaningful interpretation. Reassessment of themes was discussed until consensus was reached. A specific focus on change guided the coding and the final creation of the themes. Considerations of how time has impacted on participants’ responses, as well as attention to differences in participants’ worldviews were discussed [18]. The study was reported according to the Consolidated Criteria for Reporting Qualitative Research (COREQ) checklist criteria for reporting qualitative research [19].

## 3. Results

The themes and codes with quotations and changes over time are reported in Table 1.

### 3.1. Searching for Answers and Sense Making

At 24 months, some parents (mostly males) still struggled to make sense of the suicide, although this theme was not as dominant as observed at 6 and 12 months. Searching for answers, however, was still present. Both mothers and fathers engaged in the same contemplative processes encountered at 6 and 12 months: they spoke about the problems leading to the suicide (reporting past suicide attempts and diagnoses of mental health problems) and questioned what they could have done differently. Some parents had tried to assume their child’s perspective, envisioning the suicide as a drastic resolution to stop the pain. In one case, a parent explained how his version of the reason for the suicide differed from that of the doctors, claiming that the use of drugs, and not bipolar disorder, was the main determinant of suicide. In a few cases, parents seemed to have constructed their own personal explanation over time.

Parents did not directly blame others as often as they did in the previous months. Their narrations were more characterized by disappointment towards professionals and the perception of them as not being effective or reliable, resulting in a lack of trust in medical professionals for their own health problems (both physical and psychological). In one case, a father said that he did not hold the counselor accountable for the death of his child. Similarly to blame, anger was also not encountered as often as in the previous interviews.

After two years had passed, even those parents who did not expect the suicide of their child no longer reported shock and bewilderment. In addition, the frustration of trying to obtain information from coroners or mental health professionals was not observed. The most common emotional condition was depression: feeling emotionally worse close to the anniversaries of the death and birthdays and during the holidays. However, it was observed especially in fathers that, with the worsening of their mood, their feelings of being overwhelmed increased. Some fathers reported feeling surprised to still be feeling “down” after two years and were worried to be “still not back” to how they were before the suicide. Both mothers and fathers described having intrusive thoughts about their child, not related to the search for answers or guilt, but rather, more general and systematic.

Both mothers and fathers referred to another preoccupation not encountered earlier: the fear of others starting to forget their child. Parents described situations in which friends had trouble remembering how long ago the loss occurred, and in some cases reported how their other children appeared to talk less about their deceased sibling. One participant looked back at how the family coped immediately after the loss and reported regret at having discarded all of the daughter’s belongings.

A new desire to become emotionally closer to the rest of the family was also noted. Parents reported being preoccupied with the safety and well-being of their other family members. They spoke about being concerned for their children’s financial security or recent aggressive behaviors that had sometimes led to suicidal attempts. Fathers, specifically, spoke of the negative impact of the suicide on their partners, with one describing how he believed taking antidepressants had changed his partner’s personality. These accounts generally suggested that males believed their partners, rather than themselves, had been more impacted and were more in need of help.

### 3.2. Coping Strategies and Support

Several adaptive and maladaptive coping strategies were observed: some similar to those encountered previously. Avoidance of discussing the death was still present (for females and males), although several parents described feeling able to disclose more about the death than previously. Parents reported being able to talk about the death with relatives and friends but had occasional difficulties with their partners and significant struggles with their children. Avoidance through excessive working was still observed in males. Another maladaptive coping strategy was described by one woman, who reported binge gaming as what she believed was her only available resource for socializing and keeping her days full.

Drinking excessively was still a recurrent behavior in males; however, some reported they had significantly reduced their alcohol consumption in order to improve their health. Somatic dysregulations, such as difficulties in sleeping, were observed, but the subsequent use of drugs or alcohol to induce sleep (as previously described) were not reported. A number of health issues, related to the age of the participants, had appeared in the last year or seemed to have been aggravated. Parents who were dissatisfied with health professionals reported their own non-compliance with their practitioners’ directions, in some cases being hostile towards the use of prescribed antidepressants, and reluctant to seek help from a mental health professional when feeling overwhelmed. Parents whose children had used antidepressants prior to their suicide said they wanted to avoid what they perceived as negative effects, or declared that they believed the pharmacologic treatment was ineffective because it had led to their child’s death.

Another maladaptive coping mechanism that emerged for the first time was the belief in paranormal events involving signs from the deceased child. One father talked of how he and his wife approached a medium, in an attempt to contact their child, and two mothers described events in which they believed they could hear the presence of their child or had received messages from them.

Numerous examples of adaptive coping strategies and new positive behaviors were reported. The most frequently observed was self-care: parents reported starting to take better care of their physical health (exercising more and reducing alcohol consumption) and, in several cases, spoke about their new ability to make time and space for themselves, especially closer to the anniversary of their loss. Some parents described how being able to listen more to their own needs had a positive impact on different levels of their life; for example, several fathers decided to leave their job and find a less stressful occupation. Others reported taking up enjoyable hobbies that helped to distract them during the day, and several described a generally more positive attitude towards life’s events.

Parents described the benefits of their faith and participating in their church’s social life. Some reported becoming closer to their church after having distanced themselves soon after the loss, and described how the group helped them to keep a routine and obtain informal support. Parents also reported new rituals of memorialization, carried out by their child’s friends, for example, erecting a commemorative plaque and creating a video memorial. Continuing a personal relationship with their child through keeping a journal or writing letters was described by some fathers, who reported this allowed them to maintain a direct, intimate, and authentic relationship with the child, to whom they wrote using the same tone and attitude they used when they were alive. The journal was also considered a testimony to show to children and grandchildren. Some parents reported regretting some of the actions they adopted as coping strategies in the early months after their loss, such as cleaning the child’s room and throwing away most of their belongings. In contrast, another parent reported the action of tidying the child’s room and keeping only the most personal belongings as a step closer towards acceptance and a new connection with the surviving children, who had chosen some important personal items to keep, such as their deceased sibling’s t-shirts.

Within the family environment, some participants still report frequent conflicts with their partner; with some couples having divorced since their loss. Some parents spoke about their difficulty in reconnecting their relationships with their surviving children, who do not want to discuss the death of their sibling. In one case, a mother reported feeling her living child directly blamed her for the suicide of her brother. Overall, compared to the previous interviews, some parents believed that the loss has brought the family closer together, describing how siblings have strengthened their relationships and maintained more regular contact with their parent/s.

Lastly, experiences with professional support through attending individual counseling and/or support groups were rarely reported. Some participants said they had ceased going to individual counseling or do not consider it useful. However, two mothers reported the benefits of support groups, where they described feeling understood and finding insights through their own personal posttraumatic growth.

### 3.3. Finding Meaning and Purpose

Although acceptance of the loss was previously observed, several parents stated that their attitudes had changed in the last year and that they now felt more accepting towards their loss. Several fathers described being at peace with what had happened. Parents who were able to accept their loss talked about putting their guilt aside and being able to forgive themselves and others, realizing they had no control over the events but could control how they react to the situation. Some participants found acceptance by not dwelling on their loss and the reasons for their child’s suicide and instead respecting their child’s memory.

Some parents also described how they had reframed their loss experience as a learning opportunity. They described feeling more able to listen to themselves, and to prioritize their own self-care. For some, this process started soon after their loss and was described as a “wake-up call”. One father explained how allowing himself to be inspired by his child’s attitude had helped him to become more adventurous and, in a way, learn from his own child after the loss. Those parents who described feeling acceptance also allowed themselves to make positive decisions regarding their lives, such as undertaking travel or new experiences. Some parents were able to reconstruct the impact of the suicide and to incorporate all of that in a new narration that allows them to keep their relationship with their child untouched while still looking forward. However, it should be noted that some parents described how they were still struggling with their grief and how it would take time for them to heal.

## 4. Discussion

This unique study is a longitudinal qualitative analysis to examine the individual experiences of parents bereaved by suicide over time by analyzing changes in participants’ responses at 24 months comparing the key themes found at 6 and 12 months after their loss. To the best of our knowledge, this is the first longitudinal qualitative study of suicide-bereavement, and as such, provides important insights into the complex trajectories of grief experienced by parents over the first two years after the loss of their child to suicide.

Searching for answers and struggling to make sense of their loss was most dominant in both mothers and fathers soon after their loss, as well as was noted throughout all waves of the study. However, at two years, parents showed clearer signs of shifting from brooding to a more reflective and deliberate rumination [20]. A number of parents struggling at 6 and 12 months had developed their own personal explanation for the reasons for the suicide at 24 months. According to Moore and colleagues [8], people bereaved by suicide engage in a back-and-forth process between brooding and sense-making until they have found an explanation for the suicide that is acceptable and makes sense for them. Overall, more parents, especially males, stated that they had found acceptance of the loss of their child, suggesting that more individuals at two years are able to reach the sense-making component of the rumination phase [8]. It is possible that the diminishing emotional turmoil of the early months after the loss may have resulted in more space and time to elaborate or ruminate over the reasons for the suicide, highlighting the importance of this time period in the bereavement process.

After two years, fathers and mothers did no longer explicitly express anger as much as at 12 months. A strong polarization among parents who were still oscillating in brooding rumination and those who have shifted towards sense-making could be observed. The former more frequently reported depression symptoms, whereas the latter reported a more positive attitude towards life and acceptance. According to Stroebe and Schut [9], while women may deal with their grief with a more loss-oriented attitude by showing grief and talking about the loved one, men tend to be more restoration-oriented by engaging in social activities and mastering new skills. This attitude, not clearly observable at 6 or 12 months, was now clearly distinguishable at 24 months after the loss. In the present study, males were struck by the realization that they were still feeling overwhelmed, especially closer to important anniversaries, and described how they were “still not back” at what they were before their loss. Moreover, the observation that some parents are struggling in the same way after two years confirms the notion that some individuals may keep struggling for years after their loss and that this is likely to be more so for males [21].

It is important to note that a number of new issues were first observed at 24 months. After two years, parents were afraid of others starting to forget their children, especially the siblings. Moreover, parents frequently reported preoccupations regarding other family members and a general attempt to reconnect with them. Fathers, especially, showed a protective stance towards their partners and often suggested that their partners may need more support than themselves: this is consistent with the suggestion that females may tend to express their grief more than males [22]. In contrast to other longitudinal studies with bereaved parents [13], in this study, parents reported attempts to reconnect with their surviving children. Some parents expressed concerns about their children’s aggressive behaviors or suicide threats, and others had more general concerns about their future, their financial situation or the desire to have more regular contact with their children. Discussing the suicide of a sibling appeared to be challenging even after two years, as the parents in this study encountered hostility or avoidance when attempting to raise the topic.

In some parents, the attitude towards professionals had changed, to the point where some provided a different explanation for the death of their child to the one offered by professionals. The lack of trust in health professionals was explicitly displayed by some parents by a reduction in help-seeking attitudes or unwillingness to comply with medical treatments, either for a physical or a mental health related problem. According to the literature [23], traumatic loss also brings about health issues. For example, a longitudinal study with bereaved parents showed elevated levels of health complications at 2 and 5 years [24].

Consistent with Stroebe and Schut’s dual process model and previous findings [9,16], some parents were observed to oscillate between avoidance and restoration throughout the three observations at 6, 12, and 24 months. Some avoidant maladaptive coping strategies, such as excessive working, especially in males, have remained. Although excessive drinking was observed at 6 and 12 months after their loss, at 24 months, several parents reported trying to reduce their alcohol consumption. In addition, more parents demonstrated that they had been practicing self-care through examples, such as leaving a stressful job and making more time for themselves.

An adaptive coping strategy recurring across all observations was memorialization of their loved one. In the first months after the loss, parents engaged in behaviors aimed at keeping memories alive and maintaining a relationship with their child, such as celebrating their birthdays or keeping a journal [16]. Recent positivist models of parental bereavement place emphasis on the importance of the continuation of the bond with deceased children [25,26]. The different coping strategies encountered at 24 months, such as regret for having thrown away the child’s belongings in one case, and reporting having been able to manage a step further by tidying up the child’s room reflect, in a sense, the difference between the ruminative brooding and the ability to move towards acceptance. The strong desire to maintain a relationship with the child was also revealed in several parents through the avoidant coping strategy of the belief in paranormal experiences [27].

The social withdrawal observed in the first year after the loss appears to have been replaced by a renewed interest in rebuilding lost ties with informal groups in the second year. Informal support offered by family, friends, or groups is an important resource for survivors [28], and participation in informal groups, such as church or mutual-aid-groups, fosters a sense of purpose.

Parents’ attempts at finding meaning and purpose continued at 24 months after the suicide of their child. Their stories consistently indicated the process through which they arrived at a positive transformation, showing their ability to reorient themselves in a dramatically changed world without the deceased child, and even able to consider the loss as an opportunity to learn something new about themselves. Those parents who at 24 months were not yet able to find meaning in relation to the loss of their child, instead, showed signs of depression.

Despite the loss of a child to suicide being a “shattering” traumatic event, suicide bereavement could, in some cases, promote posttraumatic growth [8]. The literature suggests that personal growth in bereaved individuals can be found in the first year after the loss and then after an initial drop observed again and more consistently several years later [29]. Our results suggest that, at 24 months, there is a notable acceleration for some in personal growth (especially regarding self-care) but also a clearer differentiation between those who are having major difficulties in making sense of their loss. Although parents showed an improvement in their sense-making strategies at 24 months, only a few parents appeared to have arrived at a personal growth, and this may have been aided by higher resilience or a natural tendency towards positive thinking.

The main strength of our study was the application of purposive sampling, which is an important method in qualitative research to ensure representation across a range of variables. The qualitative longitudinal design of the research was particularly important in capturing and highlighting the processes involved in changes over time for suicide-bereaved parents.

The current study is limited to the parental experience of suicide bereavement and as a qualitative study has limited generalizability. It is also important to note that 3 parents (2 mothers and 1 father) were not contactable for the 24-month interviews. Nevertheless, the remaining participants still composed a good representation of mother and fathers, gender of the child, and age of parent and child, which is important in a qualitative analysis.

## 5. Conclusions

The study presented important unique processes of maternal and paternal suicide bereavement over two years after their loss. Results highlighted the importance of supporting parents in their reconstruction of social ties, especially with surviving siblings, who may be particularly struggling with their loss.

Practitioners should be informed of the potential lack of trust and non-compliance in parents who have experienced a loss to suicide, especially with regard to antidepressant treatments. All professionals involved in postvention activities should consider a more proactive approach in checking on bereaved parents’ psychological state even after two years. Moreover, practitioners should consider the need to address also other children in the family environment together with grieving parents. Overall, professionals working in the areas of social work and mental health should be further educated on the unique difficulties and specific needs of suicide-bereaved parents which may facilitate more opportunities for making sense of their loss and potential personal growth.

The oscillations between the sense-making and meaning-making phases observed at 6, 12, and 24 months confirm that the dual process model is an appropriate representation of the adaptation processes of bereavement as a dynamic and fluctuating process. The results from observations over 24 months provides hope that parents bereaved by suicide, if supported with appropriate strategies, may be able to find acceptance of their loss and positive adaptive pathways towards personal growth.

Future research should focus on further investigating gender differences among bereaved parents, as well as exploring the experiences of bereaved families, including parents and siblings.

## Figures and Tables

**Table 1:** Themes and codes at different waves of the study (Please note that pseudonyms have been used instead of real names).

	Codes	Quotations
**Theme 1. Searching for answers and sense making**		
Codes observed at 6, 12, and 24 months	*Search for answers and struggle to make sense*	No I don’t feel resentment. I just question it, “why”? In a way, I don’t blame him because it’s something that he obviously had to do in his own mind and I couldn’t read his mind, so that one I can’t really answer. (P11, Mother)
	*Past problems leading to suicide*	When he’d been at school and he’d struggled with quite a few things about being part of school, and he’d talked about harming himself at some point, then we roped in the GP and we got him to see a young person’s kind of counsellor. (P9, Father).
	*What could have been done differently*	We felt, you know, we were shut out, and we weren’t able to help… You never really get the answers, if you could have done things differently. A lot of the time, we were also frustrated that we didn’t know what the right path to take was. (P10, Mother)
	*Blame*	We all feel that the medical people let us all down, in that they didn’t help John. Afterwards, I had an interview with his doctor, and his psychologist, and I’ve been to Centrelink to talk to them, to try and let them know how important it was that they find ways of supporting and helping and connecting and liaising with each other. No one seems to talk to each other. (P10, Mother)
	*Attitude towards professionals*	I kept arguing that he was a drug user before he was bipolar, that’s the same symptoms. Finally, the doctors agreed with me just before his death… but (initially) the doctors, they were saying “no”, that bipolar came first and the drugs came afterwards. No, the drugs came first and the bipolar came second. So, (I) don’t have a lot of time for mental health (experts). (P7, Father) I guess I was thinking “well, however open or closed Archie is to this new counsellor, it’s not her fault what’s happened”. She’s done whatever she’s done, and that’s not a question I was thinking. I wasn’t really looking to apportion blame to [anybody], and I don’t still don’t. (P9, Father)
	*Anger*	I get really, really angry over it with being in a family that has had to live with suicide. I get really, really angry when people play with it. (P6, Mother)
	*Depression*	There was the funeral date and then three days later was his birthday. I was really pleased when all those dates were out of the way… It was a pretty tough time. (P4, Mother)
		But we spent winter in C… and it was terrible. I became very depressed… almost depressed enough to have to go and seek help. Yeah. Really, really, really bad times. I’m not sure what the catalyst was. I don’t know if it was Susan or it was work or it was both, but she definitely played a significant role in it, because she was on my mind a huge amount of time, and I was just sinking. Oh, it was terrible. The grey sky, the weather. (P2, Father)
	*Intrusive thoughts*	It’s a thing I still constantly see when I’m asleep and I think of it every day while I’m awake … I’ve got his twin here with me and just the things that (he’s) been doing are exactly the same as (his deceased brother). I’m going to cry. (P11, Mother)
Codes not observed at 24 months	*Shock and bewilderment*	-
	*Frustration at trying to obtain information*	-
New codes observed at 24 months	*Personal explanation*	I reckon he made a silly mistake, but in hindsight I could see that he was thinking about it for a while. He’d had a few drinks that night, and he was a bit depressed, and he had been for a little while. Yes, it was raining. I think that’s another thing when it’s raining and storming, people get depressed. Yes, I think he just made the silliest mistake of his life… he felt depressed and he wanted to do something, I’m not blaming him for it. (P8, Father)
	*Concern of being overwhelmed*	I was thinking about it, when we were going to do the next survey, and I thought, I don’t know what I make of it, but she—we had a significantly bad time, which surprised me. […] But I… but I’m still not back to as I was. (P2, Father)
	*Fear of others starting to forget*	We’ve got photos of him everywhere. But he’s definitely disappearing. I can see it now where he’s disappearing from everything… I guess people don’t talk about things like that, but I love talking about him. My wife does too, we always bring up his name, but the kids don’t, they hardly ever bring up his name. (P8, Father) I’ve had the sort of issues with the fact that I didn’t keep anything of hers, because when we were in the house and clearing up, and I thought, you know what? I don’t want anything. My granddaughter wanted lots, and that’s fine. But I didn’t keep anything at all… I’m sort of feeling bad about that, that I didn’t (keep her belongings). (P2, Father)
	*Preoccupations for other family members*	She’s very stuck is she in the experience … which makes it hard. Jamie always said that he would never do what he did because he’d break his mother’s heart. Yeah, it did… (and there are) probably other issues, as well, but I think our main issue is antidepressants. Ever since she started those, she’s been a different person. (P7, Father) I was having trouble with my kids, my elder ones. They kept threatening that they were going to do it (attempt suicide) and then one of them did but he got cut down. (P11, Mother)
**Theme 2. Coping strategies and support**		
Codes observed at 6, 12, and 24 months	*Avoidance of the topic*	A lot of people ask me what happened, that sort of thing… if I’m not ready for it, I just tell them “I’m not talking about it, we don’t talk about that”. I don’t go and push it or anything like that. I’ve got to be prepared to talk about it before I’ll talk about it. (P3, Father) I like talking about it, my wife likes talking about it, but the kids don’t. I don’t know [what’s going on with them]. I think when I was talking to them about it, they’d say they don’t want to remember sad times. I think that’s what it is, that they’d rather try not talk about him because it’s sad. (P8, Father)
	*Excessive working*	It’s not paid work but I’m always busy doing something and that’s the way I operate. (P7, Father)
	*Drinking excessively*	I can come home, and I get very, very depressed, very much like my son. If I come home and I have two drinks, and I feel—I love living, I love life, so happy. But then I just, that experience, I just like having a few more and a few more and a few more. I just want people to hurry up and get home so we can eat so I can stop drinking basically, that’s really what I do… I don’t really understand that, but it makes me feel very, very happy, I’m telling you. The next day, yes… I have been a bit depressed in the mornings, especially with the work I’ve got and whatever. (P8, Father)
	*Difficulties in sleeping*	Dissatisfied [with the quality of sleep], I wouldn’t go to very yet because I’m not cranky about it but it’s becoming a bit of a worry. (P6, Mother)
	*Health complications*	My psychiatrists and all that have written letters and saying that they think that I should be on a pension at the moment because I’ve got other things wrong with me, as well. Like broken both my wrist so I can’t really do too much work with my wrists. I’ve got curvature of the spine, I have sciatica, I get vertigo. (P11, Mother)
	*Self-care*	There’s kind of two dates that stick out in my calendar, one is the day he died and the other is his birthday. Both occasions of the two years, I’ve taken a day off work if I’ve been at work, if it’s not a weekend, and I’ve taken a drive off to the beach or up to the hills, and I suppose wanted to not do the normal stuff. So, I wanted to mark the occasion by breaking out of the ordinary routine, and I wanted to take time to reflect on where things have got to. That’s been a really positive thing. (P9, Father)
	*Positive attitude*	I’d say being active, I’ve always been positive. It’s the same… if you’ve had a bad day you just go home and [unclear] that you enjoyed yourself and you’ve had a wonderful day… If you don’t believe it, start again. (P7, Father)
	*Memorialization*	I talk to him, like I’m writing. I don’t know what you call it, but I’ve got a book and I write, and I talk to him quite often in the book. I’ve said all that to him, but it just seems like you’re really going now, no one is sort of, not cares about you, but you’ve sort of disappeared now, yes, you’re off on your own little journey type thing… that is the best thing. The two great things that I’ve felt I’ve done, one is that, and the other one is going to church and reading the bible. (P9, Father)
	*Faith*	I’ve been a fence sitter; I’ve always been a fence sitter. I used to take the family to church when they were young and they enjoyed that, but I’ve always been a fence sitter. But since he went I thought, hang on a minute, even if it’s not true, I want to believe in it. I’ve fallen off the fence to the god side. I’m going to church once a week, they’ve actually got me stuck into moving the chairs for them twice a week, because you know how the church like get people to help them and all that sort of stuff. But now I’m the chairman of church, because I move the chairs twice a week. (P8, Father)
	*Informal support*	I feel very supported by friends through that time immediately after Chris died, and then since, and still ongoing, has been a huge benefit for me. This last week, I caught up with one of my friends who I used to work with. (P9, Father)
	*Family*	He (son) says “I’ll talk about it when I’m ready” and he gets angry so I just back off. (P11, Mother) I think Sam’s death has brought the two boys closer and also our son down in Victoria. We hear from him just about every day. (P4,Mother)
	*Professional support*	You know where a psych and all that, they just read your brain and I know what’s in my head and talking to them isn’t going to get it out. (P11, Mother)
Codes not observed at 24 months	*Practical support*	-
	*Withdrawal*	-
New codes observed at 24 months	*Non-compliance & non seeking for help*	Rick was on them... and I saw what it did to him and how he always said to me “they make me feel sick, I feel horrible, I don’t want to do anything”, and he had them changed several times. (P11, Mother)
	*Beliefs in paranormal*	She only sees Stuart when her and I get on the phone, he goes to her because he can communicate through her. She’s teaching me how to be open to him and he’s told her, he actually spoke through her the last time we spoke. (P6, Mother)
**Theme 3. Finding meaning and purpose**		
Codes observed at 6, 12, and 24 months	*Acceptance*	We’ve all gone through feelings of responsibility and feeling guilt, but the guilt more now, we understand, and we realise that it wasn’t our fault, that we were all responsible as a family for each other, and that maybe we did let John down, but we can’t change it now, and it was his choice, and his life, and we didn’t push ourselves to controlling it. (P10, Mother)
	*Loss as a learning process*	I thought “well I’m going on a new adventure. I’m embracing the Archie”, which is, do something that you haven’t done before. Do something that pushes yourself to prove that you can step outside your comfort zone and actually do something. So, I was literally inspired by Archie’s example. It’s ridiculous, isn’t it, that someone who’s nearly 60 should be inspired by someone who was 21 at the time. (P9, Father)
	*Loss as a wake-up call*	I’d become a bit more lackadaisical about things generally. That’s not to say I didn’t plan holidays, didn’t do outings, didn’t do the housework, but it just felt like I had become very apathetic about things. So, Archie’s death was really like a wakeup call. Not because, I guess he’s gone, life’s changed, I have to do things differently because he’s not here. (P9, Father)
	*Impact of suicide*	I probably do that because of Peter. I wouldn’t have done it otherwise, I don’t think. (P3, Father)
	*Look forward*	Archie has died and is not with us physically, and we’re not together as a couple, but looking forward, and I guess I’ve described the kind of world I’m embracing and seeing around me now, and it’s very positive and progressive I suppose. It’s quite a helpful thing to think that you can understand it looking backwards, but you can’t live it looking backwards. You’ve got to live it looking forwards. (P9, Father)
New codes observed at 24 months	*Being at peace*	It’s much more positive than that. Which is how I really feel about his life and its influence on me. I feel very fortunate in that, because I’m sure lots of people don’t have that. (P9, Father)
	*Maintain a relationship with the child*	To me, he is still alive, in my heart he is still alive, he is still there, he is still my child. I’ll just never see him again. To me, it’s just like he’s gone on holiday or he has moved overseas. There’s no guarantee that when your children move away that you’re ever going to see them again. (P6, Mother)

## Data Availability

The data presented in this study are available on a reasonable request from the corresponding author. The data are not publicly available due to their confidential nature.
